# Functionalized Fullerene Nanomaterials: Evaluating Heteroatom Identity for Enhanced Charge-Transfer and Reactivity

**DOI:** 10.3390/molecules31071076

**Published:** 2026-03-25

**Authors:** Abdullah M. S. Alhuthali, Khaled S. Amin, Hanan Elhaes, Medhat A. Ibrahim

**Affiliations:** 1 Department of Physics, College of Science, Taif University, P.O. Box 11099, Taif 21944, Saudi Arabia; a.alhuthali@tu.edu.sa; 2 Physics Department, Faculty of Science, Al-Azhar University, Cairo 11651, Egypt; amins3981@gmail.com; 3 Physics Department, Faculty of Women for Arts, Science and Education, Ain Shams University, Cairo 11757, Egypt; hanan.elhaes@women.asu.edu.eg; 4 Spectroscopy Department, National Research Centre, 33 El-Bohouth St., Dokki, Giza 12622, Egypt; 5 Center for Converging Sciences and Emerging Technologies (CoSET), Benha National University (BNU), El-Obour 13518, Egypt; 6 Molecular Modeling and Spectroscopy Laboratory, Centre of Excellence for Advanced Science, National Research Centre, 33 El-Bohouth St., Dokki, Giza 12622, Egypt

**Keywords:** fullerene, C_60_ functionalization, DFT, B3LYP/6-31G(d,p), MESP, QTAIM, NCI, TDOS

## Abstract

This study explored the electronic and structural tunability of fullerene (C_60_) derivatives via functionalization with heteroatoms (O, S, Se) in mono-, di-, and tri-bridged configurations, including covalently modeled dimers. Calculations were performed using density functional theory (DFT) at the B3LYP/6-31G(d,p) level. Electronic descriptors such as total dipole moments (TDMs), HOMO–LUMO energy gaps (ΔE), global reactivity descriptors, total density of states (TDOS), molecular electrostatic potential (MESP) and non-covalent interactions (NCIs) were analyzed to elucidate how functionalization alters reactivity and stability. Key findings indicate that TDM increases and ΔE decreases in all functionalized C_60_; for example, the TDM increased from 0 Debye for C_60_ to 2.156 Debye for C_60_–O–S–Se, and ΔE decreased from 2.762 eV (C_60_) to 2.532 eV (C_60_–Se), indicating enhanced reactivity. This aligns with global reactivity descriptors such as reduced ionization energy and hardness. Mapped MESP surfaces showed activation around heteroatom sites. Quantum theory of atoms in molecules (QTAIM) and NCI analyses revealed that while mono-bridged structures retain covalent linkages, dimeric systems such as C_60_–O–C_60_ and C_60_–S–C_60_ relax into weak, van der Waals-type interactions. OPDOS (overlap population density of states) highlighted antibonding character between the fragments in the conduction region. These results demonstrate that heteroatom functionalization enhances the electronic properties of C_60_, making it a promising candidate for optoelectronic, organic photovoltaic, and sensor applications.

## 1. Introduction

Since their discovery in 1985, fullerenes, especially C_60_, have attracted considerable attention due to their unique cage-like structure, exceptional symmetry, and tunable electronic properties. Composed entirely of sp^2^-hybridized carbon atoms arranged in pentagonal and hexagonal rings, C_60_ exhibits a closed-shell, spherical geometry that endows it with high electron affinity, a moderate bandgap and chemical versatility [1,2,3]. In addition, fullerene has antioxidant and radical-scavenging properties [4], as well as low toxicity [5]. The characteristics render fullerenes and their derivatives appealing for utilization in the domains of material science [6,7,8], photovoltaics and electronics [9,10], drug delivery [11], cosmetics [12], pollutant gases [13], gene therapy [14] and cancer treatment [15].

Molecular modeling, particularly density functional theory (DFT), can elucidate electronic properties and possible interactions and mechanisms for molecular systems. Recent applications illustrate DFT’s broader utility; it aids in analyzing sensor interactions and mechanisms to predict and rationalize the behavior of experimental sensors [16,17,18]. It is also applied in the design of genistein–hyoscyamine composites aimed at functional-food additives identifying optimal candidates for mono-symptomatic nocturnal enuresis-targeted foods [19]. Likewise, in energy-storage materials, DFT has been used to study nitrogen and sulfur co-doped microporous carbon spheres (NS-MCSs) in flexible supercapacitor applications [20].

Building on the intrinsic tunability of C_60_, numerous DFT investigations have shown that heteroatom doping and surface functionalization can dramatically modulate its electronic landscape. For example, a recent DFT-D study demonstrated that sulfur and silicon doping not only improve the structural integrity of C_60_ but also enhance charge-transfer characteristics compared to the undoped cage, leading to increased electrical conductivity [21]. Nitrogen-doped C_60_ exhibits higher electrocatalytic activity and superior electronic features, with analogous enhancements observed upon sulfur or oxygen doping [22,23]. At interfaces, oxygen adsorption on C_60_/metal substrates has been reported to significantly alter electronic coupling and band structure, tuning charge-transport pathways and narrowing the HOMO–LUMO gap [24].

Moreover, introducing five-membered heterocycles into donor–acceptor frameworks markedly reduces the HOMO–LUMO gap, as shown by DFT/TD-DFT studies of eight ring systems supported by NICS and NBO analyses, which elucidated aromaticity and charge-delocalization trends [25]. Precise selenium functionalization of non-fullerene acceptors likewise yields red-shifted absorption, compact π-stacking, and balanced charge-transport, yielding organic solar cells with efficiencies up to 18% [26]. DFT investigations of favipiravir binding to BN-doped C_60_ report high adsorption energies and facile charge-transfer, highlighting strong B–O coordination for antiviral delivery platforms [27]. C_60_, B_12_N_12_ cages, and Al/Si-doped analogues have also been investigated for dacarbazine sensing, with boron nitride frameworks showing superior adsorption and band-gap reductions conducive to selective detection [28]. Lastly, chalcogen-modified graphene/fullerenes (O, S, Se) modelled for zidovudine delivery exhibit chemisorption energies and minimal energy gaps, favoring drug loading and mobility [29].

Despite extensive research on monomeric fullerene derivatives, dimeric systems such as C_60_–X–C_60_ (X = O, Se) remain less explored, especially in terms of their stability and bonding character. This study constructed ten fullerene derivatives to study the effects of chalcogen bridging (O, S, Se) in mono-, di-, and tri-bridged configurations. The selection of the chalcogen series (O, S, and Se) in mono-, di-, and tri-bridged configurations provides a systematic framework to investigate how atomic size and electronegativity modulate the fullerene’s electronic environment. Oxygen was chosen for its strong inductive effects, while sulfur and selenium provide larger, more polarizable electron clouds that facilitate orbital redistribution and enhanced reactivity [30,31]. Ultimately, this work aims to elucidate how heteroatom identity and bridging complexity influence the electronic and intermolecular properties of C_60_, establishing a theoretical basis for tuning these derivatives for applications in molecular sensing and optoelectronics.

A comprehensive DFT investigation was conducted at the B3LYP/6-31G(d,p) level to evaluate the total dipole moment (TDM), frontier molecular orbitals (FMOs), and global reactivity descriptors. Total density of states (TDOS), including partial density of states (PDOS) and overlap partial density of states (OPDOS), and maps of the molecular electrostatic potential (MESP) were computed. In addition, quantum theory of atoms in molecules (QTAIM) and non-covalent interaction (NCI) analyses were also employed to characterize the nature of bonding and intermolecular forces within these systems. The findings provide insights into how functionalization of C_60_ modulates fullerene’s reactivity and inter-cage connectivity, with implications for designing advanced fullerene-based nanomaterials. Such modifications highlight potential applications in fullerene-based gas sensors and electronic devices.

## 2. Results and Discussion

### 2.1. Optimized Structures

**Figure 1:**
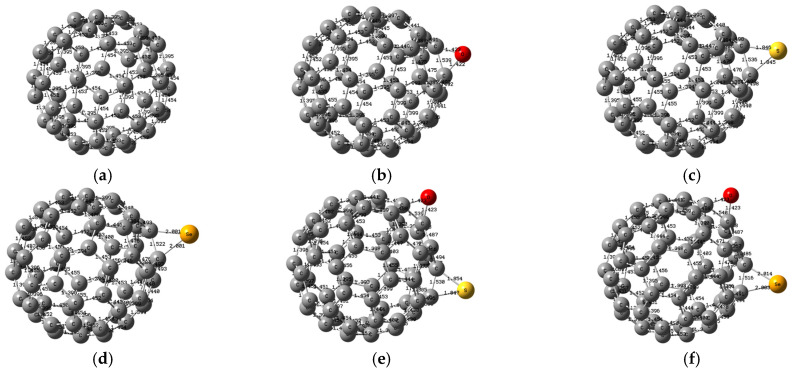

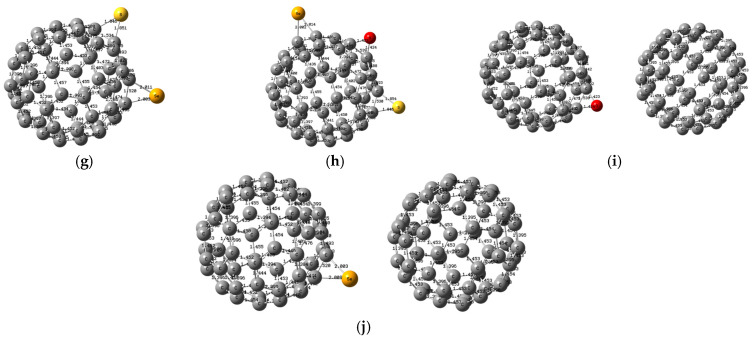
The optimized structures for the studied models: (**a**) C_60_, (**b**) C_60_–O, (**c**) C_60_–S, (**d**) C_60_–Se, (**e**) C_60_–O–S, (**f**) C_60_–O–Se, (**g**) C_60_–S–Se, (**h**) C_60_–O–S–Se, (**i**) C_60_–O–C_60_, and (**j**) C_60_–Se–C_60_.

The optimized studied structures which were calculated at the B3LYP/6-31G(d,p) level are presented in Figure 1. The geometry optimization of all C_60_ derivatives revealed distinct C–X bond lengths and C–X–C angles that reflect the size and ring strain introduced by each heteroatom linkage, presented in Table 1. In the single-site series, C_60_–O exhibits a C–O bond length of 1.422 Å and a C–O–C angle of 65.484°, whereas C_60_–S shows a longer C–S bond at 1.846 Å with a corresponding angle of 49.177°. The selenium analogue, C_60_–Se, further stretches to 2.001 Å and narrows its C–Se–C angle to 44.701°. Mixed-bridge systems introduce two distinct linkages: C_60_–O–S combines C–O (1.423 Å, 65.452°) and C–S (2.014 Å, 48.831°) bridges, while C_60_–O–Se pairs C–O (1.423 Å, 65.543°) with C–Se (2.014 Å, 44.369°). The C_60_–S–Se derivative mirrors these trends with C–S at 1.846 Å (49.014°) and C–Se at 2.011 Å (44.516°). In C_60_–O–S–Se, all three bonds coexist: C–O at 1.424 Å (65.443°), C–S at 1.854 Å (48.852°), and C–Se at 2.014 Å (44.390°). For the dimeric architectures, C_60_–O–C_60_ features a C–O length of 1.423 Å and C–O–C angle of 65.412°, with the second ‘bond’ to the neighboring cage extending to ~3.501 Å and an angle with the second cage of 22.348°. The selenium-linked C_60_–Se–C_60_ shows a C–Se bond of 2.009 Å and a C–Se–C angle of 44.546°, and an angle with the second cage of 23.637°, paired with an inter-cage separation of ~3.363 Å. These calculated parameters quantify how heteroatom identity and linking topology distort the fullerene framework, leading to alterations in its electronic properties.

### 2.2. Calculated Electronic Parameters

#### 2.2.1. Total Dipole Moment and HOMO–LUMO Energy Gap

The total dipole moment (TDM) and HOMO–LUMO energy gap (ΔE) were calculated at the B3LYP/6-31G(d,p) level for all C_60_ derivatives and listed in Table 2. A higher TDM and a corresponding lower ∆E indicate the reactivity of the studied structures [16]. C_60_ exhibits perfect symmetry with a zero TDM and a ΔE of 2.762 eV, confirming its non-polar character and serving as an ideal baseline for evaluating symmetry-breaking upon functionalization. The introduction of O, S and Se bridges breaks this symmetry, where the epoxide-like C_60_–O derivative has a TDM of 1.283 Debye and ΔE of 2.619 eV, while the sulfur and selenium analogues (C_60_–S and C_60_–Se) display lower dipoles (0.869 Debye and 0.676 Debye) and correspondingly narrower gaps (2.567 eV and 2.532 eV). Adding two heteroatoms further enhances molecular polarity. The C_60_–O–S reaches a TDM of 2.006 Debye with a ΔE of 2.604 eV, whereas C_60_–O–Se and C_60_–S–Se maintain substantial TDMs (1.850 Debye and 1.516 Debye) alongside reduced ΔE (2.575 eV and 2.570 eV). For the structure C_60_–O–S–Se, the TDM reaches 2.156 Debye, but ΔE increases slightly to 2.735 eV. Finally, the dimeric assemblies, C_60_–O–C_60_ and C_60_–Se–C_60_ exhibit moderate values of TDM (1.468 Debye and 0.271 Debye) and ΔE values of 2.594 eV and 2.516 eV. These trends demonstrate that heteroatom functionalization systematically breaks the inherent symmetry of C_60_, creating permanent dipoles that facilitate intramolecular charge polarization. The inverse correlation between increased TDM and reduced ΔE across most derivatives suggests that symmetry disruption simultaneously enhances both polarity and reactivity.

#### 2.2.2. Frontier Molecular Orbitals (FMOs)

**Figure 2:**
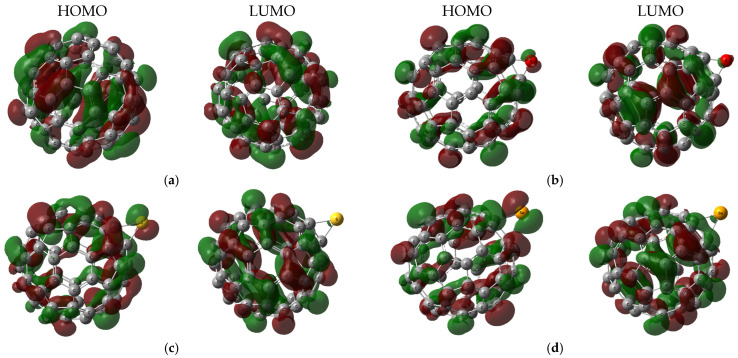

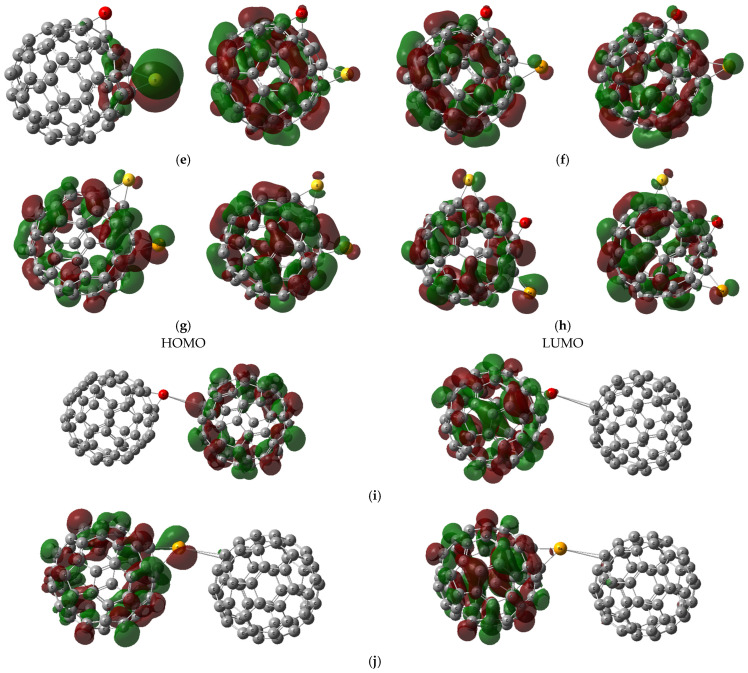
Calculated HOMO–LUMO frontier orbitals for the studied model structures showing the HOMO (**left**) and LUMO (**right**) for each derivative: (**a**) C_60_, (**b**) C_60_–O, (**c**) C_60_–S, (**d**) C_60_–Se, (**e**) C_60_–O–S, (**f**) C_60_–O–Se, (**g**) C_60_–S–Se, (**h**) C_60_–O–S–Se, (**i**) C_60_–O–C_60_, and (**j**) C_60_–Se–C_60_.

HOMO–LUMO frontier orbitals (FMOs) were calculated for the C_60_ and its functionalized derivatives at the B3LYP/6-31G(d,p) level of theory and are presented in Figure 2. Most structures exhibit broadly delocalized HOMO and LUMO distributions across the entire C_60_ framework, reflecting the uniform π-network of fullerene. A redistribution of FMOs is observed across all functionalized C_60_ systems, signifying electronic modifications. The most pronounced FMO redistribution is in the following cases: For C_60_–O–S, the HOMO becomes concentrated on the sulfur bridge, while the LUMO remains on the oxygen-functionalized cage. For the C_60_–O–C_60_ dimer, the HOMO resides predominantly on the fullerene farthest from C_60_–O, whereas the LUMO localizes on the oxygen-bridged partner, indicating asymmetric charge distribution between the two cages. For the C_60_–Se–C_60_ dimer, both the HOMO and LUMO are distributed on the cage nearest the Se atom. These localized FMO distributions establish directional charge-transfer pathways in heteroatom-modified fullerenes, offering a route to tailored electronic behavior.

#### 2.2.3. Total Density of States (TDOS)

**Figure 3:**
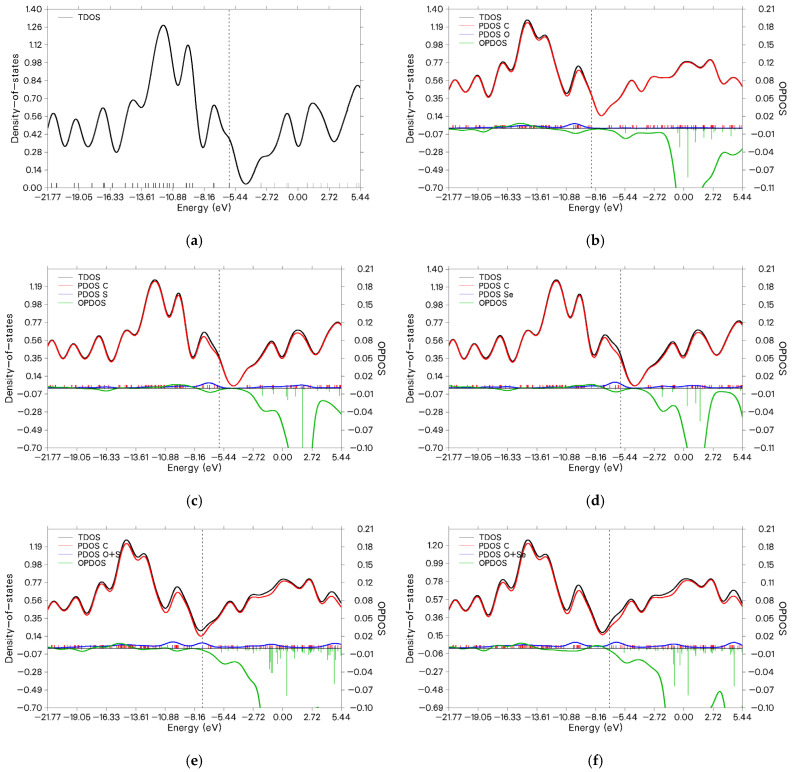

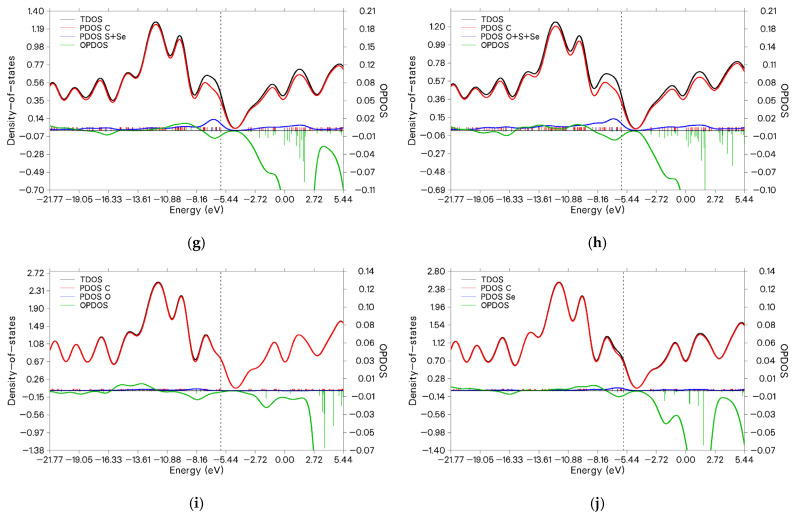
Calculated TDOS for the studied model structures: (**a**) C_60_, (**b**) C_60_–O, (**c**) C_60_–S, (**d**) C_60_–Se, (**e**) C_60_–O–S, (**f**) C_60_–O–Se, (**g**) C_60_–S–Se, (**h**) C_60_–O–S–Se, (**i**) C_60_–O–C_60_, and (**j**) C_60_–Se–C_60_.

The total density of states (TDOS) for C_60_ and its derivatives was calculated at the B3LYP/6-31G(d,p) level of theory. TDOS analysis provides insight into the distribution of electronic states as a function of energy, revealing how molecular modifications affect the electronic structure. Figure 3 displays the TDOS, partial density of states (PDOS), and overlap population density of states (OPDOS) for all studied systems. In these plots, the continuous curve represents the TDOS, constructed from the distribution of molecular orbital energy levels. Discrete vertical ticks indicate individual molecular orbitals, with black for occupied and gray for unoccupied states. A dashed vertical line denotes the HOMO energy. The PDOS, shown in red (carbon atoms) and blue (heteroatoms: O, S, Se), highlights the contributions of different atomic fragments to the electronic structure, while the green line represents the orbital overlap (OPDOS). OPDOS is particularly useful for distinguishing bonding characteristics, where positive OPDOS values indicate bonding interactions between fragments. Negative OPDOS values denote antibonding interactions. Zero OPDOS corresponds to nonbonding character. Notably, the highest contribution from heteroatoms is observed in C_60_–O–S–Se (Figure 3h), as evidenced by the prominent blue PDOS peak near the Fermi level. All systems exhibit bandgaps consistent with the ΔE values from orbital analysis. It should be noted that the non-zero TDOS observed between the HOMO and LUMO levels arises from Gaussian broadening (FWHM = 0.3 eV) applied during the plotting process in Multiwfn, which can artificially smooth and overlap discrete orbital levels, particularly in systems with narrow energy gaps. Negative OPDOS values in the conduction region signify antibonding character between specific fragments (heteroatom and C_60_). In sensor applications, adsorption of analyte molecules or photoexcitation can populate these antibonding states, potentially weakening or cleaving the heteroatom–C_60_ bond. This process may lead to a measurable change in conductivity or facilitate sensor regeneration [32,33].

#### 2.2.4. Global Reactivity Descriptors

Building on the analysis of the TDM and ΔE, based on Koopmans’s theorem, the global reactivity descriptors, ionization potential (I), electron affinity (A), chemical potential (μ), hardness (η), softness (S), and electrophilicity index (ω), were evaluated for all C_60_ derivatives. These quantities, defined by Equations (1)–(6) and calculated from the HOMO and LUMO energies obtained at the B3LYP/6-31G(d,p) level, are recorded in Table 3. For C_60_, the ionization energy was 5.987 eV, which decreased upon functionalization, with C_60_–Se and C_60_–Se–C_60_ exhibiting the lowest values of 5.840 eV and 5.804 eV, respectively. This indicates that less energy is required to ionize the modified cages. Chemical potential (μ) values ranged from −4.546 eV (C_60_–Se–C_60_) to −4.672 eV (C_60_–O–S). Electron affinity (A) and electrophilicity (ω) also increased for the dual-bridged derivatives C_60_–O–Se and C_60_–O–S relative to those for C_60_ (ω reached as high as 8.418 eV for C_60_–O–Se), signifying an enhanced tendency to accept electrons. Absolute hardness decreased and softness increased for all derivatives, pointing to greater chemical reactivity; this correlates with the previously observed TDM and narrower ΔE. The functionalization of C_60_ notably alters its electronic properties, generally leading to lower ionization potentials, increased electron affinities and electrophilicity, decreased hardness, and increased softness, thereby enhancing the overall chemical reactivity of the fullerene derivatives compared to that of C_60_, consistent with trends reported in previous studies [34].

### 2.3. Molecular Electrostatic Potential (MESP)

**Figure 4:**
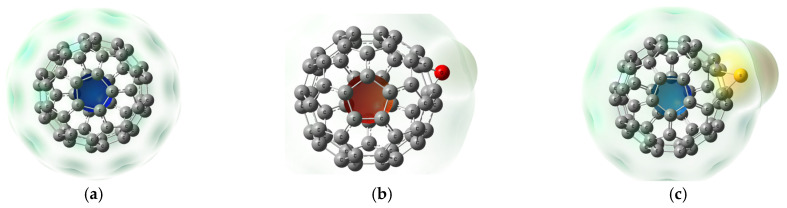

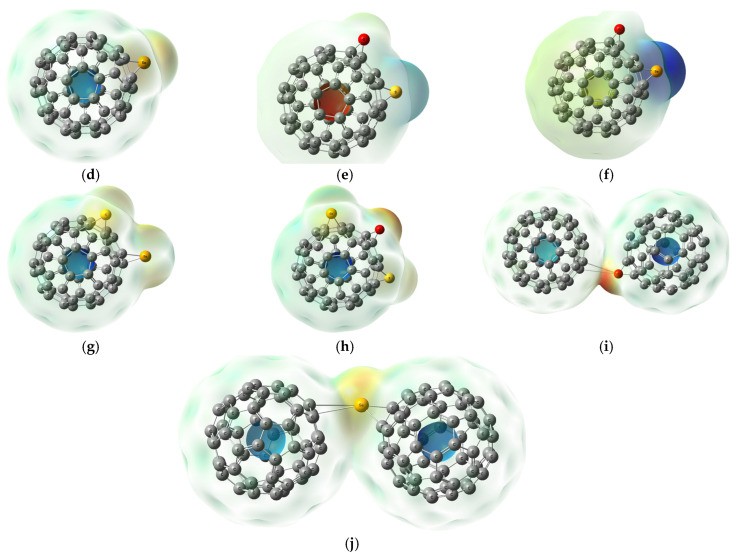
Mapped molecular electrostatic potential (MESP) for the studied structures: (**a**) C_60_, (**b**) C_60_–O, (**c**) C_60_–S, (**d**) C_60_–Se, (**e**) C_60_–O–S, (**f**) C_60_–O–Se, (**g**) C_60_–S–Se, (**h**) C_60_–O–S–Se, (**i**) C_60_–O–C_60_, and (**j**) C_60_–Se–C_60_.

The molecular electrostatic potentials (MESPs) were computed at the B3LYP/6-31G(d,p) level. MESPs can show the active sites of molecules, indicating the positive and negative potential regions by a color code [35,36]. The color ranges from red to blue, where red represents a negative region and blue is a positive region of electrostatic potential, while green color indicates a more neutral region. Figure 4 presents the MESP surfaces for all studied derivatives. The differences in the electrostatic potential distribution are mainly related to the electronegativity and atomic size of the heteroatoms. Oxygen, being more electronegative than sulfur and selenium, attracts electron density more strongly and therefore generates a more pronounced negative potential region. In contrast, the lower electronegativity and larger atomic radii of sulfur and selenium lead to a weaker localization of electron density, resulting in relatively more positive or less negative electrostatic regions around these atoms. In case of C_60_ (Figure 4a), the surface is largely neutral with a slight positive potential at the core. Epoxidation (C_60_–O, Figure 4b) inverts the core to negative (red). Sulfur- and selenium-functionalized cages (C_60_–S and C_60_–Se, Figure 4c,d) develop a subtle negative potential (yellow) around the heteroatom while the core remains positive but weakened. The mixed O–S derivative (C_60_–O–S, Figure 4e) shows slight positive potential around the S atom, and the core has negative potential, whereas the O–Se analogue (C_60_–O–Se, Figure 4f) exhibits a strong positive region around Se and slight negativity at the core. The C_60_–S–Se structure (Figure 4g) retains the same heteroatom-centered negativity seen in the mono-functionalized cages. In C_60_–O–S–Se (Figure 4h), negative potential is localized around O, with the remainder of the surface similar to that of C_60_–S–Se. Finally, the dimers C_60_–O–C_60_ and C_60_–Se–C_60_ (Figure 4i,j) both display negative potential at the bridging atom (red around O and yellow around Se). Overall, heteroatom functionalization, particularly that in C_60_–O–Se, induces the greatest surface activation and suggests enhanced reactivity.

### 2.4. Topological and Non-Covalent Interaction Analysis

To understand the nature of bonding and weak intermolecular forces within the fullerene-based composites, we applied quantum theory of atoms in molecules (QTAIM) followed by non-covalent interaction (NCI) analysis with reduced density gradient (RDG) mapping.

#### 2.4.1. Quantum Theory of Atoms in Molecules (QTAIM)

**Figure 5:**
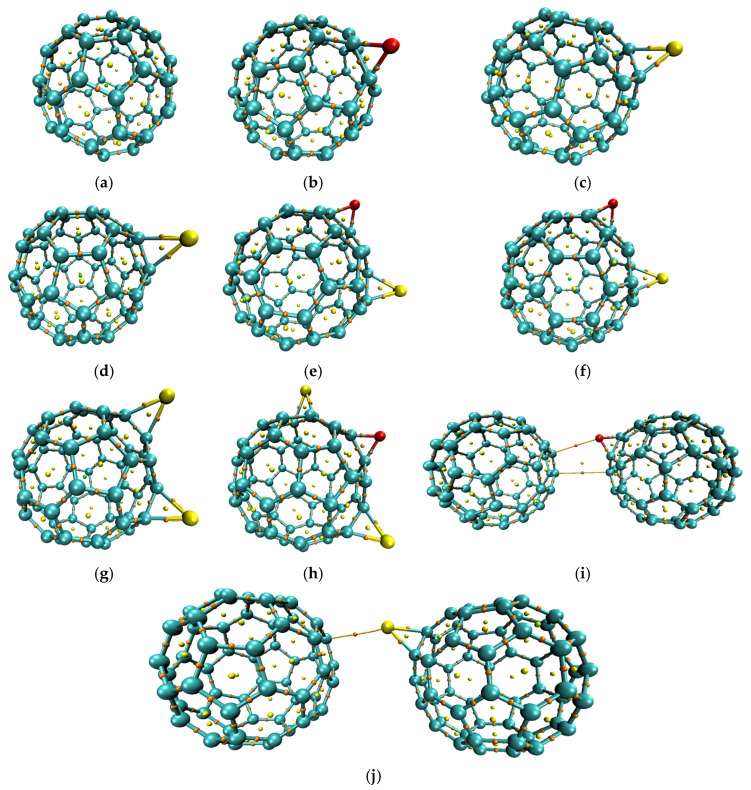
Calculated quantum theory of atoms in molecules (QTAIM) topology for the studied structures: (**a**) C_60_, (**b**) C_60_–O, (**c**) C_60_–S, (**d**) C_60_–Se, (**e**) C_60_–O–S, (**f**) C_60_–O–Se, (**g**) C_60_–S–Se, (**h**) C_60_–O–S–Se, (**i**) C_60_–O–C_60_, and (**j**) C_60_–Se–C_60_.

QTAIM uses the distribution of electronic density ρ(r) to identify important parameters like bond paths and bond critical points (BCPs) that reflect the nature and strength of interactions [37,38]. Covalent bonds typically exhibit high values of ρ(r) but negative values of ∇^2^ ρ(r) and energy density H(r). Non-covalent or closed-shell interactions, such as hydrogen bonds or van der Waals (vdW) interactions, exhibit smaller values for ρ(r), with ∇^2^ ρ(r) > 0 and H(r) > 0 [39]. Figure 5 displays the QTAIM topology for fullerene and its derivative structures showing bond critical points (BCPs) and bond paths, calculated at the B3LYP/6-31G(d,p) level. In Figure 5 C_60_ and all fullerene derivatives show stable formations with clear BCPs and bond paths indicating preserved covalent frameworks. The QTAIM descriptors further provide insight into the bonding nature between the heteroatoms (O, S, and Se) and the adjacent carbon atoms of the C_60_ cage. The presence of bond critical points between the heteroatoms and neighboring carbon atoms indicates the formation of chemical bonds within the fullerene framework. The corresponding electron density characteristics at the BCPs, including relatively high values of ρ(r) together with negative ∇^2^ ρ(r) and negative total energy density H(r), are consistent with interactions of predominantly covalent character. At the same time, heteroatom incorporation slightly perturbs the original C–C bond connecting the two carbon atoms, which is reflected in the redistribution of electron density within the local bonding environment.

However, in the dimeric structures C_60_–O–C_60_ and C_60_–S–C_60_, where covalent bonds are initially modeled (epoxide-like or thiirane-like bridges), geometry optimization leads to spatial separation between the two fullerenes. The QTAIM analysis of the C_60_–O–C_60_ structure reveals a weak interaction between the bridging O and C atom, indicated by low ρ(r) (0.041 a.u.), positive ∇^2^ ρ(r) (0.015 a.u.), and positive H(r) (0.007 a.u.), consistent with a non-covalent bond. Additionally, a secondary BCP is observed between carbon atoms of the two fullerene cages with even lower ρ(r) (0.020 a.u.). Similarly, in C_60_–S–C_60_, the BCP between the bridging S and C atom of the two fullerene cages shows low ρ(r) (0.010 a.u.), positive ∇^2^ ρ(r) (0.029 a.u.), and positive H(r) (0.001 a.u.), indicating a weak interaction in the inter-cage region. The absence of covalent BCPs and elongated inter-cage distances (~3.2–3.5 Å) further support the presence weak vdW interactions. The instability of C_60_–O–C_60_ and C_60_–S–C_60_ under geometry optimization suggests that epoxide/thiirane-like covalent bridges are thermodynamically unfavorable in dimeric systems.

#### 2.4.2. Non-Covalent Interaction (NCI) and Reduced Density Gradient (RDG)

**Figure 6:**
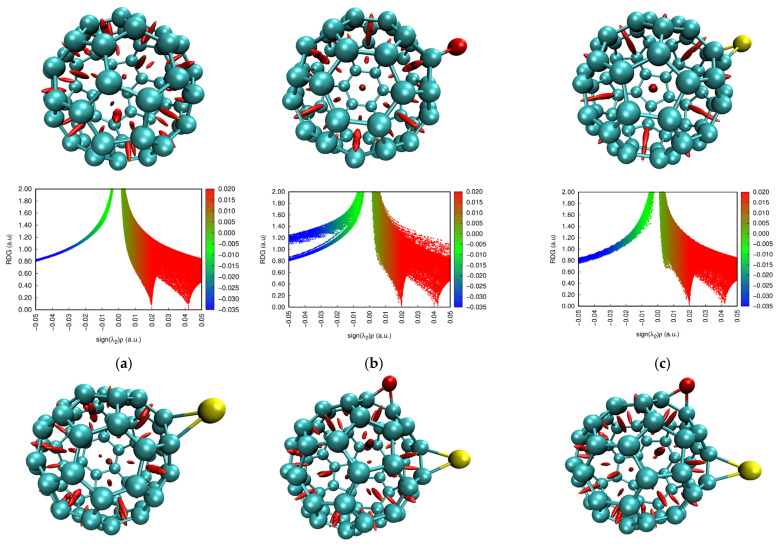

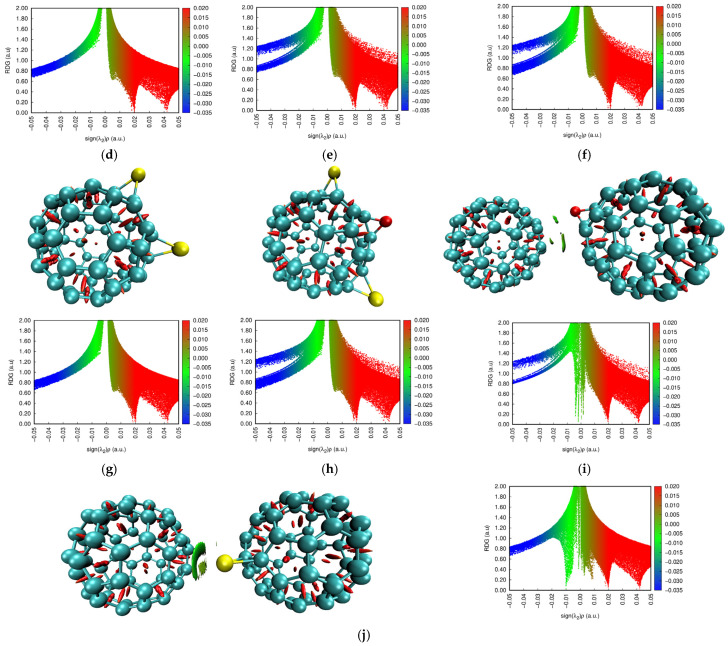
Calculated non-covalent interactions (NCIs) and reduced density gradient (RDG) for the studied structures: (**a**) C_60_, (**b**) C_60_–O, (**c**) C_60_–S, (**d**) C_60_–Se, (**e**) C_60_–O–S, (**f**) C_60_–O–Se, (**g**) C_60_–S–Se, (**h**) C_60_–O–S–Se, (**i**) C_60_–O–C_60_, and (**j**) C_60_–Se–C_60_.

**Figure 7:**
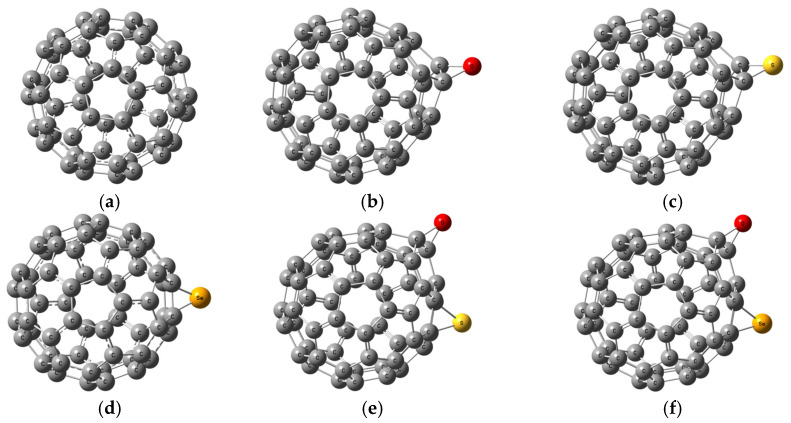

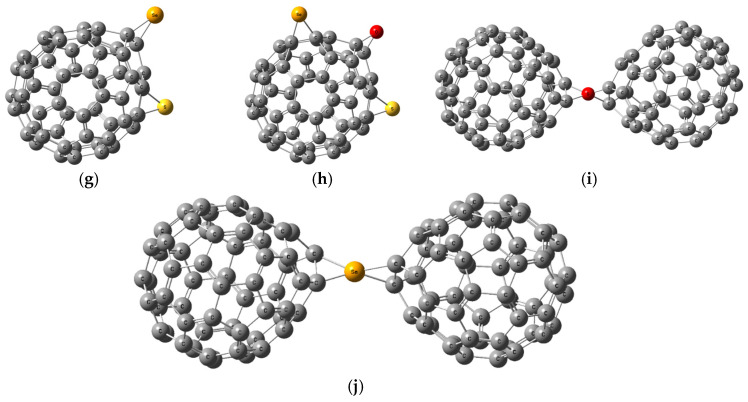
The studied model structures: (**a**) C_60_, (**b**) C_60_–O, (**c**) C_60_–S, (**d**) C_60_–Se, (**e**) C_60_–O–S, (**f**) C_60_–O–Se, (**g**) C_60_–S–Se, (**h**) C_60_–O–S–Se, (**i**) C_60_–O–C_60_, and (**j**) C_60_–Se–C_60_.

To gain deeper insight into the intermolecular interactions within the composites, NCI analysis combined with RDG mapping was conducted. These techniques enable the visualization of weak non-covalent forces such as hydrogen bonding indicated by blue color, vdW interactions indicated by green color, and steric repulsions indicated by red color [40,41]. The NCI and RDG calculated at the same level of theory and generated are shown in Figure 6. All fullerene-based structures show the typical red isosurfaces around the C_60_ cages due to steric crowding [42]. More importantly, NCI-RDG maps reveal green isosurfaces between the separated fullerenes, indicating vdW interactions. These observations are in agreement with the QTAIM results. Notably, the C_60_–S–C_60_ system exhibits stronger vdW stabilization, as evidenced by more pronounced RDG peaks (green peaks) and a larger green interaction surface compared to the C_60_–O–C_60_ counterpart. Figure 7i,j further illustrate these interactions, showing green isosurfaces between the fullerene surfaces, where S and Se interact with surface carbon atoms. These regions confirm the presence of dispersion interactions that stabilize the dimers without forming strong covalent or hydrogen bonds. The broader green region observed in the C_60_–Se–C_60_ system reflects enhanced dispersion stabilization, aligning with the QTAIM analysis and supporting the conclusion that weak vdW interactions play a significant role in the functionalized dimer geometry.

### 2.5. Binding Energies

The calculated binding energies for all investigated systems are summarized in Table 4. A clear periodic trend is observed in the monatomic functionalization of C_60_: the binding strength follows the order O>S>Se. Specifically, the C_60_–O system exhibits a robust binding energy of −6.11 eV, which significantly decreases for C_60_–S (−3.62 eV) and C_6_0–Se (−3.36 eV). This trend is consistent with the decreasing electronegativity and increasing atomic radii down the chalcogen group, which leads to a weaker covalent overlap with the carbon π-network of the fullerene cage. For the multi-chalcogen systems (C_60_–O–S, C_60_–O–Se, and C_60_–S–Se), the binding energies are substantially higher, reaching −12.87 eV for the C_60_–O–S–Se complex. This suggests a synergistic stabilization effect, likely arising from both the individual atom–cage interactions and the formation of chalcogen–chalcogen bonds on the surface. Furthermore, the bridging complexes (C_60_–X–C_60_) show binding energies comparable to those of their monatomic counterparts (e.g., −6.15 eV for C_60_–O–C_60_), indicating that the chalcogen atom acts as an effective linker between the two carbon spheres without significant loss of structural stability.

The QTAIM and RDG topology revealed non-covalent, van der Waals-type interactions rather than covalent bonding between the chalcogen and C_60_. This suggests that the chalcogen atom acts as a ‘non-covalent spacer’ rather than a chemical bridge. The high binding energy in these cases is attributed not to orbital overlap (covalency) but rather to strong dispersive forces and electrostatic polarization facilitated by the chalcogen atom between the two large electron clouds of the cages. This ‘relaxed’ non-covalent bridging explains the structural stability of the sandwich complexes in the absence of traditional chemical bonds [43,44].

### 2.6. Benchmark Comparison of Basis Sets for Chalcogen-Functionalized Aromatics

To assess the reliability of the B3LYP/6-31G (d,p) level of theory, benchmark calculations were performed on smaller model systems (benzene–O, benzene–S, benzene–Se) using the larger aug-cc-pVTZ basis set. The model structures are represented in Figure S1. The total dipole moment (TDM) and HOMO–LUMO energy gap (ΔE) was compared as shown in Table S1. The results show very good agreement between the 6-31G (d,p) and aug-cc-pVTZ basis sets. For all three systems, the calculated TDM and HOMO–LUMO gap differs only slightly between basis sets, with trends consistently preserved. This confirms that the 6-31G (d,p) basis set provides reliable qualitative and semi-quantitative predictions for chalcogen-functionalized systems, justifying its use in the main study. 

## 3. Materials and Methods

### 3.1. Calculation Details

The studied model structures were optimized using Gaussian 09 software [45], which was implemented at Molecular Modeling and Spectroscopy Laboratory, Centre of Excellence for Advanced Science, National Research Centre, Giza, Egypt. Each structure was optimized using the B3LYP functional [46,47,48], a combination of the Lee–Yang–Parr (LYP) correlation functional and Beck’s three-parameter hybrid exchange functional (B3). The 6-31G(d,p) basis set, featuring polarization functions on both heavy (d) and hydrogen (p) atoms, was used for a more accurate representation of polar bonds in molecules. The B3LYP/6-31G(d,p) level of theory was selected as a well-established approach that provides a reliable balance between computational cost and accuracy for large carbon-based systems such as fullerene derivatives, as demonstrated in numerous studies on C_60_ geometries, electronic properties, and relative stabilities [49,50,51]. All calculations were performed considering a neutral total charge and a singlet spin multiplicity. Some important parameters were calculated at the same level of theory, such as the total dipole moment (TDM) and the highest occupied molecular orbital and lowest unoccupied molecular orbital (HOMO–LUMO) band gap energy (ΔE). In addition, molecular electrostatic potential (MESP) maps were generated. Based on the values of HOMO and LUMO energy, some descriptors are called global reactivity, such as the ionization potential (I), electronic affinity (A), chemical potential (μ), chemical hardness (η), absolute chemical softness (S) and electrophilicity index (ω). These descriptors were computed with the following Equations (1)–(6) [52]:

I = −E_HOMO_ (1)

A = E_LUMO_ (2)

µ = −(I + A)/2(3)

η = (I − A)/2(4)

S = 1/η(5)

ω = µ^2^/2η (6)

The total density of states (TDOS), partial density of states (PDOS) and overlap partial density of states (OPDOS) were generated using Multiwfn software 2.0 [53]. To check the stability of the studied models, quantum theory of atoms in molecules (QTAIM) calculations were conducted using Multiwfn and visual molecular dynamics VMD software 1.0 [53,54]. Non-covalent interaction (NCI) analysis, complemented with the reduced density gradient (RDG), was also performed using the same software.

To evaluate the stability of the chalcogen-functionalized fullerene systems, binding energies (E_bind_) were calculated using the standard supramolecular approach:

E_bind_ = E_complex_ − ∑E_fragments_ (7)

where E_complex_ represents the total electronic energy of the functionalized C_60_ system, and ∑E_fragments_ is the sum of the energies of the isolated C_60_ cage and the respective chalcogen atoms (O, S, Se).

### 3.2. Building Model Molecules 

Buckminsterfullerene (C_60_) was first fully optimized at the B3LYP/6-31G(d,p) level (Figure 7a). Subsequent derivatives were modeled by inserting heteroatom bridges or inter-cage linkers, followed by full re-optimization at the same level of theory. In the single-site series, an epoxide-like O bridge across one C–C bond yields C_60_–O (Figure 7b), while replacement of O with S or Se produces C_60_–S (Figure 7c) and C_60_–Se (Figure 7d), respectively. Mixed-bridge derivatives then combine two non-adjacent linkages: C_60_–O–S (Figure 7e) carries both an epoxide and a thiirane bridge, C_60_–O–Se (Figure 7f) pairs the epoxide with a selenide bridge, and C_60_–S–Se (Figure 7g) bears both S and Se. C_60_–O–S–Se (Figure 7h) incorporates all three linkages on the cage. Finally, two dimeric architectures, C_60_–O–C_60_ (Figure 7i) and C_60_–Se–C_60_ (Figure 7j), were built from cages placed about 3.0 Å apart and then optimized to form the final O- and Se-bridged dimers.

## 4. Conclusions

The present study provides a comprehensive computational analysis, using DFT calculations at the B3LYP/6-31G(d,p) level, for the structural and electronic impacts of the functionalization of C_60_ with O, S and Se. C_60_ exhibited zero TDM and a HOMO–LUMO energy gap of 2.762 eV, whereas the tri-bridged C_60_–O–S–Se derivative had the highest TDM (2.156 Debye) and the C_60_–Se derivative had the lowest gap (2.532 eV). Other derivatives showed similar trends, underscoring enhanced polarity and reactivity. FMO analyses revealed orbital redistribution and directional charge-transfer pathways in the localized LUMO in the C_60_ derivatives. Global reactivity descriptors confirmed that functionalization lowers ionization potentials, increases electron affinity and electrophilicity, decreases hardness, and increases softness, enhancing overall chemical reactivity. PDOS showed the heteroatoms’ contribution, especially in C_60_–O–S–Se, and OPDOS plots showed negative peaks in the conduction region, indicating antibonding character. OPDOS antibonding can be utilized upon analyte adsorption or photoexcitation, which could weaken heteroatom–C_60_ bonds and yield measurable conductivity changes useful in sensor applications. MESP mapping highlighted increased surface reactivity, with C_60_–O–Se exhibiting the greatest surface activation. QTAIM and NCI–RDG analyses showed that mono- and di-bridged derivatives retain stable covalent frameworks, but dimeric C_60_–O–C_60_ and C_60_–S–C_60_ relax into weak vdW assemblies. Consistent with this interpretation, binding energy analysis indicated strong stabilization for the functionalized systems, with the strength of monatomic interactions following the order O > S > Se and multi-chalcogen structures exhibiting additional stabilization due to cooperative cage–atom and chalcogen–chalcogen interactions. Overall, the observed trends reflect a consistent relationship among the properties, where systems with increased TDM often exhibit reduced HOMO–LUMO gaps, higher electrophilicity, and enhanced surface reactivity. This alignment between electronic descriptors, orbital behavior, and non-covalent interaction patterns highlights the integrated impact of functionalization. The selected analysis techniques were chosen to offer a comprehensive view of structure–property relationships. These insights provide a solid theoretical foundation for practical exploration of the most promising derivatives, particularly C_60_–O–S–Se and C_60_–Se, in optoelectronic, organic photovoltaic, and gas sensors.

## Figures and Tables

**Table 1:** Optimized C–X bond lengths (where X is the heteroatom from left to right) and C–X–C angles for all C_60_ derivatives optimized at B3LYP/6-31G(d,p).

Structures	C–X Bond Length (Å)	C–X–C Angle (°)
C_60_–O	1.422	65.484
C_60_–S	1.846	49.177
C_60_–Se	2.001	44.701
C_60_–O–S	1.423, 2.014	65.452, 48.831
C_60_–O–Se	1.423, 2.014	65.543, 44.369
C_60_–S–Se	1.846, 2.011	49.014, 44.516
C_60_–O–S–Se	1.424, 1.854, 2.014	65.443, 48.852, 44.390
C_60_–O–C_60_	1.423, 3.501	65.412, 22.348
C_60_–Se–C_60_	2.009, 3.363	44.546, 23.637

**Table 2:** Calculated total dipole moment (TDM) in Debye and HOMO–LUMO energy gap (ΔE) in eV for the studied structures.

Structures	TDM (Debye)	ΔE (eV)
C_60_	0.000	2.762
C_60_–O	1.283	2.619
C_60_–S	0.869	2.567
C_60_–Se	0.676	2.532
C_60_–O–S	2.006	2.604
C_60_–O–Se	1.850	2.575
C_60_–S–Se	1.516	2.570
C_60_–O–S–Se	2.156	2.735
C_60_–O–C_60_	1.468	2.594
C_60_–Se–C_60_	0.271	2.516

**Table 3:** Calculated global reactivity descriptors for the studied structures derived from the HOMO and LUMO energies: ionization potential I (eV), electron affinity A (eV), chemical potential μ (eV), chemical hardness η (eV), absolute chemical softness S (eV^−1^), and electrophilicity index ω (eV).

Structure	I (eV)	A (eV)	μ (eV)	η (eV)	S (eV)^−1^	ω (eV)
C_60_	5.986	3.225	−4.606	1.381	0.724	7.679
C_60_–O	5.945	3.326	−4.636	1.310	0.764	8.203
C_60_–S	5.877	3.310	−4.594	1.284	0.779	8.220
C_60_–Se	5.840	3.308	−4.574	1.266	0.790	8.262
C_60_–O–S	5.974	3.370	−4.672	1.302	0.768	8.380
C_60_–O–Se	5.944	3.369	−4.656	1.288	0.777	8.418
C_60_–S–Se	5.923	3.354	−4.639	1.285	0.778	8.374
C_60_–O–S–Se	5.988	3.253	−4.621	1.367	0.731	7.807
C_60_–O–C_60_	5.929	3.336	−4.633	1.297	0.771	8.274
C_60_–Se–C_60_	5.804	3.288	−4.546	1.258	0.795	8.213

**Table 4:** Calculated binding energy E_bind_ in eV for the studied structures.

Structures	E_bind_
C_60_–O	−6.110
C_60_–S	−3.616
C_60_–Se	−3.361
C_60_–O–S	−9.618
C_60_–O–Se	−9.373
C_60_–S–Se	−6.887
C_60_–O–S–Se	−12.868
C_60_–O–C_60_	−6.146
C_60_–Se–C_60_	−3.526

## Data Availability

The data supporting the findings of this study can be obtained from the corresponding author upon request, subject to reasonable conditions.

## Supplementary Materials

The following supporting information can be downloaded at: https://www.mdpi.com/article/10.3390/molecules31071076/s1, Figure S1: Model molecules for benzene derivatives functionalized with O, S, and Se: a, benzene; b, benzene-O; c, benzene-S; d, benzene-Se; Table S1: A comparison of TDMs in Debye and ΔE in eV for benzene derivatives functionalized with O, S, and Se, calculated using B3LYP/6-31G (d, p) and B3LYP/aug-cc-pVTZ.

## Abbreviations

The following abbreviations are used in this manuscript:

DFT	Density functional theory
B3LYP	Becke’s three-parameter exchange functional together with Lee–Yang–Parr correlation functional
TDM	Total dipole moment
ΔE	Energy gap
HOMO	Highest occupied molecular orbital
LUMO	Lowest unoccupied molecular orbital
MESP	Molecular electrostatic potential
FMO	Frontier molecular orbital
TDOS	Total density of states
PDOS	Partial density of states
OPDOS	Overlap partial density of states
QTAIM	Quantum theory of atoms in molecules
BCP	Bond critical point
ρ(r)	Electronic density
H(r)	Energy density
NCI	Non-covalent interaction
RDG	Reduced density gradient
I	Ionization potential
A	Electronic affinity
μ	Electronic chemical potential
η	Chemical hardness
S	Absolute softness
ω	Electrophilicity index
vdW	van der Waals
C_60_	Buckminsterfullerene
