# Supplementary material for: Functionalized Fullerene Nanomaterials: Evaluating Heteroatom Identity for Enhanced Charge-Transfer and Reactivity
Source: Molecules. 2026 Mar 25;31(7):1076. doi: 10.3390/molecules31071076 (PMC13075211; doi:10.3390/molecules31071076)
Supplement: Supplementary file 1 [file molecules-31-01076-s001.zip › molecules-4195205-supplementary.pdf]

Supplementary Material

Benchmark Comparison of Basis Sets for Chalcogen-Functionalized Aromatics

To assess the reliability of the B3LYP/6-31G (d, p) level of theory, benchmark calculations were performed on smaller model systems (benzene–O, benzene–S, benzene–Se) using the larger aug-cc-pVTZ basis set. The model structures are represented in figure s1. The total dipole moment (TDM) and HOMO–LUMO energy gap (ΔE) were compared as shown below. The results show very good agreement between the 6-31G (d, p) and aug-cc-pVTZ basis sets. For all three systems, the calculated TDM and HOMO–LUMO gap differ only slightly between the basis sets, with trends consistently preserved. This confirms that the 6-31G (d, p) basis set provides reliable qualitative and semi-quantitative predictions for chalcogen-functionalized systems, justifying its use in the main study.

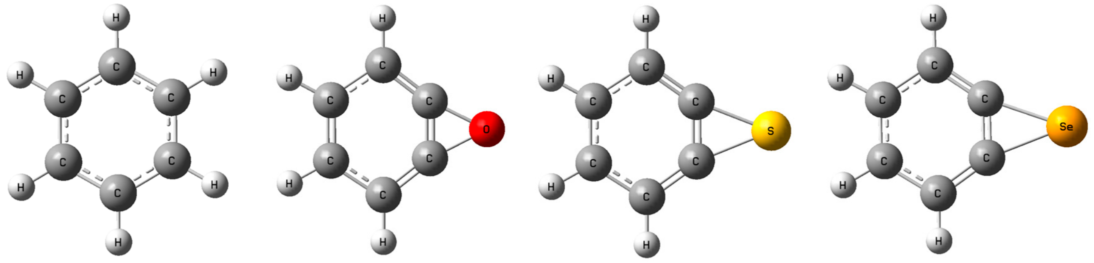

a b c d

Figure S1. Model molecules for benzene derivatives functionalized with O, S, and Se: a, benzene; b, benzene-O; c, benzene-S; d, benzene-Se.

Table S1. A comparison of TDMs in Debye and ΔE in eV for benzene derivatives functionalized with O, S, and Se, calculated using B3LYP/6-31G (d, p) and B3LYP/aug-cc-pVTZ.

Structure	TDM (Debye)		ΔE (eV)	
	6-31G (d, p)	Aug-CC-pVTZ	6-31G (d, p)	Aug-CC-pVTZ
**Benzene**	0.000	0.000	6.791	6.612
**Benzene-O**	1.899	1.877	5.671	5.517
**Benzene-S**	1.702	1.485	4.777	4.752
**Benzene-Se**	1.442	1.393	4.300	4.230
